# Insights into the Social Determinants of Health in Older Adults

**DOI:** 10.4236/jbise.2022.1511023

**Published:** 2022-11-02

**Authors:** Felipe P. Perez, Carmen A. Perez, Magali N. Chumbiauca

**Affiliations:** 1 Department of Medicine, Division of General Internal Medicine and Geriatrics, Indiana University School of Medicine, Indianapolis, IN, USA;; 2 School of Social Work, Indiana University, Indianapolis, IN, USA;; 3 Richard L. Roudebush Veterans Affairs Medical Center, Home Based Primary Care, Indianapolis, IN, USA

**Keywords:** Social Determinants, Integrated Plan, Older Adult, Health, Personalized, Social Services

## Abstract

In this paper, we review the social determinants of health in older adults and their complex interrelationship with medical diseases. Also, we provide recommendations to address these determinants in the integrated healthcare plan. The social determinants in older adults and its influence in health outcomes have been studied for decades. There is solid evidence for the interrelationship between social factors and the health of individuals and populations; however, these studies are unable to define their complex interrelatedness. Health is quite variable and depends on multiple biological and social factors such as genetics, country of origin, migrant status, etc. On the other hand, health status can affect social factors such as job or education. Addressing social determinants of health in the integrated healthcare plan is important for improving health outcomes and decreasing existing disparities in older adult health. We recommend a person-centered approach in which individualized interventions should be adopted by organizations to improve the health status of older adults at the national and global level. Some of our practical recommendations to better address the social determinants of health in clinical practice are EHR documentation strategies, screening tools, and the development of linkages to the world outside of the clinic and health system, including social services, community activities, collaborative work, and roles for insurance companies.

## 1. INTRODUCTION

Several studies have tried to identify how social and structural determinants of health impact the health and social care needs of patients. Some of these studies lack standardization of what variables define the social determinants of health and the appropriate screening tools to track these variables, inconsistent data and measurement, and inadequate healthcare-based solutions for the core problems such as access to care, poverty and food insecurity [1].

Medical providers are trained in biological determinants of health however may lack knowledge in the implications of social factors that impact health outcomes. Common medical studies do not delve into the social determinants of health. Interestingly, a recent study found that the relative contributions of social determinants of health on wellbeing outcomes were around 60% [2].

It is a priority for health care providers to know the effects of these social determinants on patient’s health care outcomes (Table 1). A comprehensive understanding of the impact of social determinants on health can ultimately improve health in individuals and populations as clinicians can offer more effective treatments, improved social screening, timely referrals to legal and social services, and initiation of research to understand the mechanisms by which social factors affect health.

**Figure 1.**
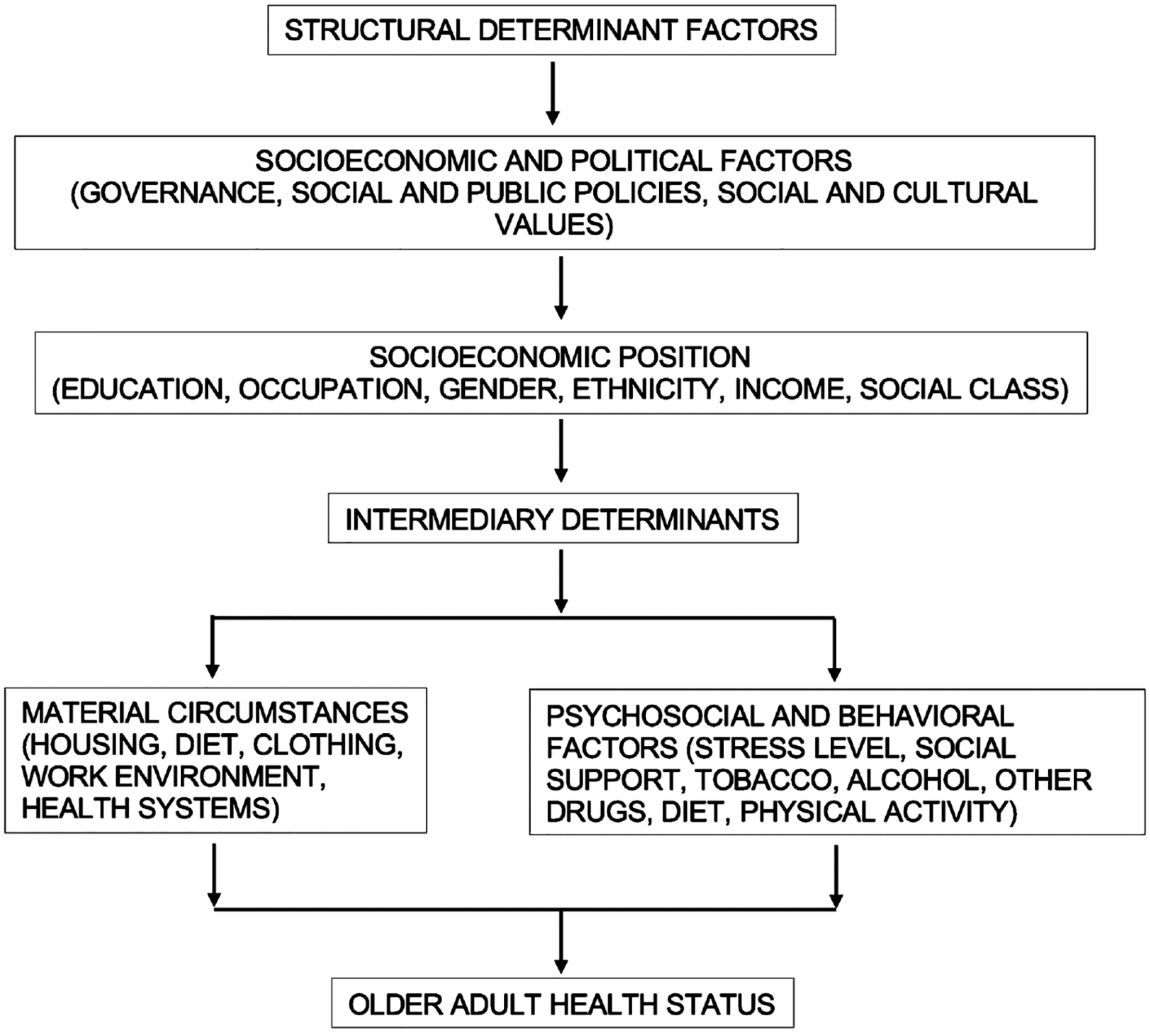
Sequence of the effects of the social determinants of health on the older adult health status.

Understanding that structural determinant factors [3] (governance, social and public policies, social and cultural values) determine an individual’s socioeconomic position (education, occupation, gender, ethnicity, income, social class) which affect the intermediary determinants such as material circumstances (housing, diet, clothing, work environment, health systems) and the psychosocial and behavioral factors (stress level, social support, tobacco, alcohol, other drugs, diet, physical activity) helps unravel the complexity of the interaction of all of these factors at many levels (Figure 1).

The aim of this paper is to review the literature on social determinants and its’ impact on the health of older adults in the United States and to develop a conceptual framework for clinicians to better understand the importance of social determinants on the health of older adults.

## 2. RACE AND ETHNICITY DETERMINANTS OF HEALTH AT OLDER AGE

Racial and ethnic inequalities have been associated with adverse health outcomes [4]. Life expectancy is a parameter to measure health and will be discussed. In the United States, years of life expectancy at birth in non-Hispanic Blacks is 74.9 in men and 78.1 in women is the lowest. In whites it is 78.7 in men and 81.1 for women. In Hispanics it is 81.8 and 84 in men and women respectively. The higher life expectancy for Hispanics seems to be a paradox considering that Hispanics have a lower socioeconomic status, education, and health care resources than the white population. Despite poorer household incomes, poor access to health care, and worse health [5]. Hispanics have a longer life expectancy than white people. Boen found Hispanics have higher disability, depressive, metabolic, and inflammation compared to Whites. Boen did not find a healthier status in Hispanic immigrants. Interestingly socioeconomic factors are important determinants of inflammation, disability, depressive, metabolic, as these differences are attributed to stress exposure [6].

Furthermore, US- and foreign-born Hispanics had lower physical function and depression than Whites. Also, foreign-born Hispanics have the same metabolic syndrome risk as White. Conversely, US-born Hispanics have increased risk of metabolic syndrome than Whites. Lucas found that among the Hispanic subgroups Puerto Ricans had poorer health state (19.2%) than Mexicans (17.4%), Cubans (14.7%), and Central or South American (12.3%) [7]. Also, Puerto Ricans had additional multiple chronic diseases, increased psychological distress, and were more likely to be unable to work due to health problems that the other Hispanics subgroups.

Studies also found that Hispanics and African Americans health risk was very similar. Further the disparity between foreign-born Hispanics and Whites declined with age, offering evidence of the age-as-leveler hypothesis. However, this contradicts the fact that Hispanics live longer than whites. Therefore, there must be other factors that cause the increase life expectancy in Hispanics. One of these factors may be that Hispanics have strong communities and often live in multigenerational households that care for one another. Even though morbidity is high in adult life, they are taken care of by family members. Also, Hispanics have a higher life expectancy due to good health behaviors, the healthiest come to the United States living in communities where they have strong support systems. Interestingly, it is important to note that life expectancy in Hispanic countries is lower that the Hispanics in the United States which raise questions about social or biological factors that impact life span such as differences in health care systems.

## 3. EDUCATION

Cutler and Lleras-Muney estimated the basic correlations between education and health by the following formula [8]:

Hi=c+βEi+Xiδ+εi

where *Hi* is an individual *i*’s health, *Ei* stands for individual *i’s* years of education, *Xi* is the individual characteristics including race, gender and single year of age, *c* is a constant term and ε is the error term. The coefficient on education *β* (education gradient) is the object of interest, and it measures the effect of one more year of education on the patient’s health.

The study’s results show that individuals with higher levels of education are less likely to die within 5 years. They also found that the more educated also report having lower morbidity from the most common acute and chronic diseases (heart condition, stroke hypertension, cholesterol, emphysema, diabetes, asthma attacks, ulcer). The only exceptions are cancer, chicken pox and hay fever.

Education can play a factor in improving health outcomes for individuals while diseases can decrease your chances of obtaining a better education. Interestingly, Singh found that higher education significantly increases life expectancy in older Whites (19 years) and African Americans (21 years) however not in Hispanics (18 years) [9]. Thus, there must be other social factors that counteract the positive effect of education in older Hispanics. One factor could be the Hispanics familism defined as a stronger orientation toward the family [10], in which acceptance and love to all their family member regardless of their educational achievements would decrease psychological stress and increase wellbeing [11].

Additionally, lower education has been associated with diabetes and hypertension [12]. However, education contributed to lower mortality from diabetes in Hispanics [13], making the analysis of these factors more complicated. Furthermore, Jemal *et al*. found that possibly preventable determinants associated with lower education could be the associated with a great proportion of all deaths in US adults [14].

## 4. TYPE OF WORK

Occupation has been associated to health outcomes and life expectancy. In a recent study they found that white-collar men and women with no activity limitations between the ages of 50 and 75 had a life expectancy of about 4.5 years longer than in low skilled blue-collar occupations [15]. High skilled blue-collar and low skilled white-collar occupations without activity limitations had a 2.3- and 3.8-years shorter life expectancy, compared to high skilled white-collar men. Low skilled white-collar women without activity limitations had a life expectancy of 2.6 years shorter than high skilled white-collar women.

## 5. INCOME AND WEALTH

Wealth has been associated with better health status and higher life expectancy in multiple studies. Research of the causality between health and wealth indicates a bidirectional relationship between health and wealth. In 11 countries, a one-way causality was found as being from wealth to health and in 8 countries the other way around. Also, a two-way relationship was observed in 2 countries [16].

## 6. YOUNG ADULT LIFE EXPERIENCES AS DETERMINANTS OF HEALTH IN OLDER AGE

The interaction between young adult life experiences and older adult health has only recently begun to attract attention to researchers [17]. Healthcare providers should review the patient’s life course for the understanding of this interaction. Healthcare providers should examine the individual experiences and the affect with on future medical comorbidities, as each change over time and in their interaction with external historical conditions.

Life course events such as exercise, happiness, stress, social support, health care, early age at marriage, high parity, and experience of adverse events, such as the death of a child and being dismissed from work has been associated with health in early old age [18].

## 7. LATE LIFE EXPERIENCES AS DETERMINANTS OF HEALTH IN OLDER AGE

### 7.1 Social Aging

#### 1) Retirement in older age.

There is an obvious relationship between poor health and early retirement [19]. However, it is unknown how an early retirement affects health in older adults. For a long time, it was thought that retirement was detrimental to older adult health [20]. However, a European study found that retirement decreases the probability of reporting to be in fair, bad, or very bad health; subsequently it has a positive effect preserving general health [21].

#### 2) Psychological and physical effects

Aging can cause physical symptoms such as fatigue, pain, and weakness which can produce poor health inhibiting functioning and decreased social engagement [22]. These adverse physical symptoms can provoke isolation and loneliness which typically increases in older adults [23].

In a recent study, Kotwal found that the overall prevalence of social isolation and loneliness in older adults was 1 in 5 and increases prior to death [24]. However, in another study more than 60% of older adults rarely account loneliness and less than 10 % state severe loneliness [23].

Furthermore, loneliness has been associated to increased mortality in several studies [25, 26]. Some investigators have hypothesized several biological mechanisms that increase poor health and mortality such as cardiovascular activation, cortisol levels, sleep and health behaviors [27].

### 7.2 Work after Retirement

Post-retirement has been associated with greater psychological well-being and life-satisfaction [28]. Studies have found that individuals who work after retirement show improved physical and mental health outcomes [29]. Furthermore, Nikolova found that voluntary part-time workers are happier, experience less stress, less anger, and have higher job satisfaction than full-time employees [30].

### 7.3 Exercise

Exercise has been associated with good health outcomes such as improved cognition and preventing dementia [31]. Furthermore, Lee found an inverse linear dose-response relation between volume of physical activity and all-cause mortality [32].

### 7.4 Social Support

Married individuals have healthier physical and mental state than single individuals, however married individuals have increased risk of overweight and obesity than single individuals [33]. One explanation for this paradox is that despite a higher weight married people have healthier eating habits and behaviors [34].

Health insurance studies showed that the lack of insurance in American adults generate less appropriate healthcare, and it was associated with higher mortality among white adults who had low incomes, diabetes, hypertension, or heart disease [35].

Currently around 1.6 billion people worldwide lack adequate housing (20% of the world’s population) and, it is estimated, that annually about 2 million people are formally evicted from their homes. Novoa found that housing conditions can impact one’s physical and mental health by the emotional housing conditions, the physical housing conditions, the physical environment, and the social environment of the neighborhood [36].

Nguyen found that family and friend relationships are associated with well-being. Also, Nguyen found that qualitative aspects such as closeness and negative interaction are more important than structural aspects such as frequency of contact [37].

Seeman found that increased social relationships have positive effects such as decreased mortality in older adults; however, negative effects such as depression and angina were also found 38. These negative effects could be related to increased cortisol and norepinephrine 38. Therefore, these data suggest that the quality of social relationships determine the positive or negative effects.

### 7.5 Nutrition

Nutrition is a major factor in older adults health and human health in general [38]. Interestingly, Ordovas found that Personalized Nutrition plan motivates people to follow a healthy diet and lifestyle when compared conventional dietary advice [39].

## 8. CONCLUSION

The nation expects an increase in the number of older adults as the population ages; therefore, it is crucial to educate healthcare providers about older adults and their caregivers social care needs. It is important to address all the social determinants of health in the integrated healthcare plan of older patients to develop individual interventions such as the Personalized Nutrition plan. Healthcare organizations should adopt these interventions to improve the health status of older adults at the local, national, and global level.

There are still multiple unresolved challenges due to the extensive heterogeneity across race, culture, genetics, resources, education, and other social factors, which makes a consensus statement defining the social determinants of health in older adults difficult to approach. The fact that patient’s health issues also affect social factors makes defining causality almost impossible.

Furthermore, efforts to create a person-centered integrated healthcare plan and develop individualized protocols that address the patient’s social and health status interrelationships should be implemented in health care settings. This can be achieved by increasing screening and documentation of the social determinants of health documentation in the electronic health record. These records will allow providers to use the collected data for the integrated health care plan to make medical decision and referrals to social care services [40].

## Figures and Tables

**Table 1.** Social determinants of health from the World Health Organization (2017).

	Social determinants of health*
1	Income and social protection
2	Education
3	Unemployment and job insecurity
4	Working life conditions
5	Food insecurity
6	Housing, basic amenities and the environment
7	Early childhood development
8	Social inclusion and non-discrimination
9	Structural conflict
10	Access to affordable health services of decent quality
